# Insecure Employment Contracts during the COVID-19 Pandemic and the Need for Participation in Policy Making

**DOI:** 10.3390/ijerph182312548

**Published:** 2021-11-28

**Authors:** Maryam Maleki, Abbas Mardani, Mojtaba Vaismoradi

**Affiliations:** 1 Pediatric and Neonatal Intensive Care Nursing Education Department, School of Nursing and Midwifery, Tehran University of Medical Sciences, Tehran 1416753955, Iran; maleki-m@razi.tums.ac.ir; 2 Nursing Care Research Center, Department of Medical Surgical Nursing, School of Nursing and Midwifery, Iran University of Medical Sciences, Tehran 1449614535, Iran; 3 Faculty of Nursing and Health Sciences, Nord University, 8049 Bodø, Norway; mojtaba.vaismoradi@nord.no

**Keywords:** COVID-19, employment contract, healthcare policy, job security, nurse, workforce

## Abstract

Job security influences the ability of nurses to provide high-quality nursing care. The Iranian health system has always faced nursing shortages, and the COVID-19 pandemic has worsened this situation. Although nurses have been labelled ‘heroes’ across the globe, many of them have been hired using insecure employment contracts. This commentary aims to describe issues surrounding job contracts for Iranian nurses during the COVID-19 pandemic and discusses how the current situation can be improved. Iranian nurses are at the frontline of the fight against COVID-19 and need to receive better support in terms of job security and dignity. They should participate more in policymaking activities to improve their job condition and prevent the development and implementation of the short-term and insecure job contracts that lead to job insecurity.

## 1. Introduction

Public health has been defined as “the art and science of preventing disease, prolonging life and promoting health through the organized efforts of society” [1]. It aims to decrease health inequities among population groups and improve the health of the entire population [2]. Nurses constitute the largest group of healthcare professionals and have a vital role in the overall wellbeing and health of the population [3]. They have a longstanding and successful history of protecting and promoting public health through devising strategies aiming at the prevention of diseases [4].

The quality of work life (QWL) of nurses plays an important role in the promotion of public health [5,6]. QWL is a multifaceted concept consisting of job security, workplace conditions, job content, job promotional opportunities, sufficient and fair recompense, duty discretion, involvement in decision-making processes, occupational stress, organizational security in employment, and work–life stability [6].

Job security is defined as the employee’s perception that they can maintain their job for as long as they desire, and no mental or objective factor makes them prone to losing their job [7]. Job insecurity is the most important dimension of work in all professions and countries [8]. In the nursing profession, job security is directly associated with work engagement, job satisfaction, organizational performance, a low level of turnover, and high-quality patient care [7,9,10].

Work engagement is a satisfying and positive state of work characterized by vigor, dedication, and absorption [11]. Autonomy and trust are preconditions for work engagement [12]. The consequences of nurses’ work engagement include the reduction of mortality rates, a lower turnover of nurses, an improvement in nursing care quality, and an enhancement of the profitability of healthcare organizations [12,13,14,15]. 

Job satisfaction has been defined as the sense of pleasantness when job needs or desires are met [16]. It has been found among nurses that job dissatisfaction has a direct association with increased stress, depersonalization, turnover intention, low quality of care, and intention to leave the job [14,17,18]. Organizational performance is the success or effectiveness of an organization and is known as an indicator of how an organization performs to achieves its goals successfully [19]. Organizational performance is influenced by employee engagement and satisfaction [20,21].

Nurses’ job insecurity can reduce public health due to dissatisfaction and reduction in QWL [6]. Nurses’ job insecurity is influenced by the type of employment relationship, income, working hours, instability, right to freedom of association, and work conditions [22]. Nurses’ perceptions of job insecurity can influence their health and quality of life, predispose them to physical fatigue and psychological problems, and can reduce their effort in their work [23,24]. The results of Zhang et al.’s study showed that nurses with more job insecurity experienced more emotional exhaustion [25]. Furthermore, job insecurity can reduce workplace performance, creativity, and satisfaction with basic human needs [8].

A recent study in Spain showed that job security in nursing has worsened in the last decade, mainly due to falling numbers of permanent job contracts [26]. Another study on 32,000 nurses in 10 European countries reported that nearly half of them experienced job insecurity [27]. In a study by Burke and Singh, the feelings of job insecurity among 290 nurses in Canada were reported to be relatively low [9], while a study in Iran showed that 22% of 558 participating nurses had low job security [23]. 

In this commentary, we present an overview of the Iranian health system and the nursing profession in Iran based on a thorough review of the literature. We continue with a discussion on the condition of Iranian nurses after the COVID-19 pandemic and the employment contracts in this period. Finally, this paper concludes by proposing some directives about how nurses’ job conditions and security in Iran can be improved.

## 2. Iranian Health System

The health system in Iran is primarily an insurance-based system [28]. The Ministry of Health and Medical Education (MoHME) of Iran is responsible for implementing policies to achieve the highest level of healthcare at a national level, which are implemented by medical universities across the country. The highest health officials in the provinces throughout the country are the presidents of the medical universities. They are responsible for medical education, public health, and the provision of healthcare in public facilities [29].

The public sector provides primary, secondary, and tertiary healthcare services. Some primary healthcare services, including vaccinations and prenatal care, are provided free of charge. In addition, a significant portion of secondary and tertiary healthcare services in the provinces are provided by the public sector. The private sector also provides secondary and tertiary healthcare in urban areas. Furthermore, non-governmental organizations (NGOs) are active in certain areas such as diabetes, pediatric cancer, thalassemia, and breast cancer. The MoHME is responsible for planning, monitoring, and supervising health-related activities for private and public sectors in Iran [29,30,31].

On 5 May 2014, the Iranian health system started the implementation of the Health Sector Evolution Plan (HSEP). Its goal was to reduce the co-payment of patients admitted to public hospitals, distribute physicians to less-developed areas of the country, provide incentives to encourage physicians to stay and work in disadvantaged areas, reduce inequalities between different regions of the country, increase equality in access to healthcare services, increase the presence of specialist physicians residing in public hospitals, improve the quality of visiting services, and update healthcare tariffs. In addition, policy making in the fields of prevention and primary healthcare were planned [32,33].

Currently, due to the shortage of nursing in Iran, most nurses provide services in hospitals. This is because most health officials and those involved in the Iranian health system prefer to employ nurses in the second level of prevention, i.e., clinical care in hospitals [34,35]. However, the contribution of nurses to public health and the primary level of prevention is gradually increasing. They are more involved in the education of community health workers in community health centers [34,35,36]. At the third level of prevention, Iranian nurses have recently been working in welfare centers, rehabilitation centers, palliative care centers, and home-care centers [34,35].

Furthermore, nurses have an important role in the implementation of the HSEP. Indeed, with the implementation of the HSEP and expansion of public health coverage, and the subsequent reduction in treatment tariffs in public centers, referrals to these centers have increased. However, the number of nurses has remained stable, leading to an increased workload for nurses working in public health centers [37,38].

## 3. Nursing Profession in Iran

High-school graduates can enter the nursing program at medical sciences universities across the country based on their interests and rank in the national entrance exam [39]. Currently, university programs for nursing include bachelor’s, master’s, and doctoral degrees in nursing. To be considered qualified, nurses must obtain a bachelor’s degree in nursing and be recognized as registered nurses [40]. Most nurses working in the Iranian health system have a bachelor’s degree in nursing science [41]. The bachelor’s, master’s, and doctoral degrees in nursing are approved by the nursing board at the MoHME. This board is also responsible for validating nursing schools and revising nursing curricula [42].

Clinical placement for undergraduate nursing students begins in the second semester in teaching hospitals. Students should participate in a clinical placement until the end of their third year whilst undertaking theoretical courses. They receive training in their first three years under the direct supervision and guidance of nurse instructors. Their fourth year is dedicated to an internship, which is performed under the supervision of nurse instructors and under the direct guidance of clinical nurses [39,43]. A bachelor’s degree is the minimum requirement for nursing practice. According to law, before being eligible for employment in other healthcare settings such as private centers and home-care centers, all nursing graduates in Iran are required to work in government-run centers such as hospitals, community health centers, and palliative care centers for 2 years [44].

The nursing shortage for positions in health centers under the supervision of the Iranian health system is a major and multifaceted challenge. The causes of nursing shortages in Iran can be ordered based on priority and importance as follows: unwillingness to enter and stay in the nursing profession, insufficient salaries, low social status, negative perception of nursing being a career primarily for women, low QWL, insufficient support in the workplace, and emigration to other countries [45,46]. However, unwillingness to enter and stay in the nursing profession has been introduced as the main reason behind nursing shortages in Iran [45]. According to the World Bank, the number of nurses per 1000 population in developed countries has been over 10 during the last decade [47]. By increasing the number of nursing schools across Iran and the enrollment of nursing students during the last decade [48], the number of nurses increased from 1.4 nurses per 1000 people in 2004 to 2.6 nurses per 1000 in 2017 [47]. Nevertheless, the Iranian health system still suffers from nursing shortages. According to a report by the MoHME, 140,000 nurses are working in the Iranian health system and the ratio of nurses to patients in Iran is 0.5–0.8. In developed countries, the ratio is estimated at 1.8. Therefore, another 260,000 nurses are needed to reach the standard ratio and maintain the desired level of healthcare [37,49]. 

Proper salaries are one of the main strategies to attract and retain the workforce [50]. Payments in the Iranian health system to non-medical workforces are based on the combination of fixed salaries and performance-based payments, and nurses cannot obtain additional incomes through the provision of private services in government-run healthcare settings [51]. Studies in Iran have shown that the bulk of the healthcare sector’s expenditures in all healthcare settings is mainly dedicated to physicians [50]. However, health care depends on teamwork and team members need to receive a fair income. Fair payment improves the QWL, job motivation, job satisfaction, and the quality of care delivered to clients [50]. Low salaries for nurses, large income gap between specialized and qualified nurses and general physicians in all healthcare settings, and failure to implement tariffs for nursing services are common reasons for dissatisfaction among Iranian nurses [50,52]. 

Cultural and contextual diversities and the socio-cultural infrastructure of societies influence the social image of nursing [53]. In the Iranian context, society usually does not much appreciate the role, work, and education of nurses. Therefore, nurses do not receive enough respect [41]. They are often introduced as medical assistants rather than patient advocates, which causes them to lose their credibility [41,54]. Additionally, nurses have a low level of self-esteem, and feel confused, hopeless, and frustrated about their self-image and social identity [39]. Improper portrayal of nursing by media, poor financial rewards, and job insecurity are reasons for the creation of such feelings [53,55]. Nursing is still trying to be recognized and accepted as a profession by Iranian society [56]. 

The negative impression of nursing as a woman’s career discourages men from choosing nursing as a career. The male-to-female ratio for nurses in Iran is estimated at 1:7 [57]. One of the most influential factors preventing Iranian men from choosing the nursing profession is the social image of nursing as a woman’s career, portraying nurses as physician assistants [58]. The poor image of nursing and the low social status of nurses are the main causes of the high turnover among Iranian male nurses [57,59], which has exacerbated nursing shortages.

The findings of a recent study showed that 69.3% of Iranian nurses were dissatisfied with their work life [6]. A low QWL is attributed to reforms in the Iranian health system after the implementation of the HSEP. Inequalities in payment and heavy workloads, particularly in public hospitals, have been the consequences of such reforms [33,60]. Another major contributing factor to low QWL and job dissatisfaction are poor healthcare management and support, inattention to the needs of nurses and requests by relevant organizations, high job stress, job insecurity, lack of involvement in decision making, and unfair promotion policies [6,61,62]. In this respect, 78% of Iranian nurses reported that their managers did not reply to their concerns and complained about leadership issues in the workplace [63]. Therefore, Iranian nurses working in all healthcare settings have been gradually migrating from Iran for a long time [64]. The exact statistics on the migration status of Iranian nurses are unavailable, but the literature confirms that nurse migration is one of the main reasons for nursing shortages in Iran [45,64]. 

## 4. Condition of Iranian Nurses after the COVID-19 Pandemic

The first confirmed cases of COVID-19 were reported by the MoHME in Iran on 19 February 2020 [65]. Measures taken by the Iranian health system to control the COVID-19 pandemic have been insufficient. As of 20 November 2021, 6,073,098 confirmed COVID-19 cases were reported in Iran, of whom 128,852 died [66].

There is a common international notion that pandemics impose a substantial burden on healthcare systems, particularly on human resources [67], due to increasing referrals and hospitalization of patients [68], working with limited medical facilities and equipment, heavy workloads, and exposure to infection [69]. Nurses, as the largest group of healthcare professionals, are the key components of coordinated responses to critical healthcare situations [70,71,72].

Iranian nurses have played various roles during the COVID-19 pandemic to improve people’s health. Most of them provide direct clinical care to patients with COVID-19 in hospitals. Nurses in community health centers contribute to screening for COVID-19, contact tracing and case reporting, provision of education and consultation to the public about basic health measures, quarantine and isolation, risk management, occupational health, mental health support, and vaccination [73]. 

Iranian nurses who provide direct clinical care and health practice in hospitals or community health centers have experienced many challenges during COVID-19, including anxiety, stress, fear of being judged, frustration, worrying about self/others, feeling abandoned, physical exhaustion, living with uncertainty, and social stigma [74,75,76,77,78]. In addition, nursing shortages, insufficient protective equipment, lack of support from healthcare officials, and excessive workloads have contributed to nurses’ willingness to quit their job [75,76,77,79].

Furthermore, the COVID-19 pandemic has driven Iranian nurses to foreign countries that have offered better financial and workplace incentives. Although accurate and official statistics on the migration of Iranian nurses during the COVID-19 pandemic are not available, according to unofficial statistics provided by the Iranian Nursing Organization (INO), the migration of nurses has tripled during COVID-19 and around 100 nurses migrate from Iran every month [80]. Therefore, nursing shortages have been felt more in Iran during the COVID-19 pandemic.

The COVID-19 pandemic is still one of the most important health issues in Iran. The Iranian health system and society have been severely affected by this pandemic. According to the basics of crisis management science, planning should be done before turning an accident into a crisis as much as possible, but in relation to the COVID-19 pandemic, basic measures in the field of prevention in Iran came after the disease had already turned into a biological crisis [81].

At the beginning of the COVID-19 pandemic, the Iranian health system suffered severely from nursing shortages [82]. In response to this crisis, medical sciences universities across the country affiliated with the MoHME issued a formal call to recruit part-time nurses. This call developed insecure employment contracts characterized by an increase in the maximum hours of a part-time contract to 100 h per month, a reduction in the contract period to 89 days with the possibility of extension if necessary, the removal of the obligation for an employer to hire a nurse, the lack of social security or occupational insurance provision, and the existence of contracts outside the scope of labor law. According to Iranian labor law, working days for a full-time employee are considered all days during a week except Fridays and official holidays. Additionally, a full-time employee works 8 h per day, and the total number of working hours per week is 44 h.

After the formal call to recruit part-time nurses, many nurses were hired in different cities, especially in areas with a red COVID-19 outbreak status, based on insecure employment contracts without granting appropriate job benefits to nurses in hospitals and community health centers. In fact, several nurses who were needed during the COVID-19 crisis were recruited with very low salaries, less than half of the salary of employed nurses, and without job security, leading to job discrimination. At the end of an 89 day period, many nurses must choose to lose their jobs or accept another similar job contract [83,84]. There are no clear statistics on the number of nurses employed on 89 day contracts.

Nurses are morally and professionally obliged to provide high-quality care to patients [85]. This obligation extends to care for patients in emergency conditions such as in the COVID-19 pandemic [86]. In their oath, nurses promise that regardless of margins, patients will be at the forefront of their attention [87]. In a phenomenological study in Iran, nurses mostly declared that their professional commitment and work conscience encouraged them to continue working in the COVID-19 critical situation and attempt to cover work shifts for those nurses who became sick [88]. It seems that the professional and moral commitment of Iranian nurses has played an important role in accepting such insecure employment contracts.

Nevertheless, beyond professional and moral commitment, in the context of the COVID-19 crisis, it is unreasonable to employ nurses using inappropriate policies leading to their exploitation.

The short-term nature of this type of contract leads to job insecurity for nurses [89]. This feeling will have adverse consequences including the emigration of nurses to other countries to earn higher wages and avoid increased workloads, burnout, and stress. It also increases the vulnerability of nurses, ultimately reducing the quality of care delivered to patients and increasing mortality rates [8,22,25,89]. In addition, work conditions influence the health conditions of nurses [90]. Temporary employees experience higher insecurity, lower psychological well-being, more psychosomatic problems and provide a lower public health quality of service than permanent employees [91]. 

## 5. Directives to Improve Nurses’ Job Conditions and Security

Nursing practice in all aspects is regulated by healthcare policies and is influenced by related changes [92]. The promotion of public health is considered the ultimate goal of health policies [93]. Nurses’ participation in policy making extends the caring spirit of the nursing profession and promotes public health [2]. Nurses are valuable sources of insight and information affecting the formulation of health policies, and can prevent unintended consequences [94].

Historically, nurses have played a limited role in national policies and policy decisions affecting healthcare issues [95]. Although Iranian nurses are the largest group of healthcare staff, accounting for 65% of healthcare professionals [96], they have a limited impact on health policies [97] because the MoHME is responsible for all healthcare policies [98] and physicians are dominant in policymaking initiatives [99]. Therefore, these insecure employment contracts are not observed in the Iranian physician community. Additionally, nurses spend most of their time with patients and their families, and they are far from the policymaking table [99]. Therefore, the power of nurses in the healthcare system has not grown appropriately [100].

The nursing organization and four nursing associations in Iran, including INO, Iranian Nursing Association (INA), Iranian Cardiac Nursing Association (ICNA), and Iranian Scientific Nursing Association (ISNA), can potentially influence healthcare policies affecting the nursing profession [101]. The policymaking power and the number of members of the INO are greater than those of other nursing associations. The INO as an NGO was established in 2001 after several years of efforts by nurses. The main mission of the INO is to defend nurses’ rights, improve nursing in society, and enhance their knowledge and skills through the provision of on-the-job training [101,102]. However, it has no executive power and cannot greatly impact policies made by the MoHME affecting the nursing profession.

Healthcare systems across the globe act within the framework of healthcare policies. Nurses have the required ability and are well-qualified to participate in health policy development using their knowledge of the healthcare system, as well as their analytical and communication skills [103]. In order to improve their work and professional status and prevent exploitative employment contracts, Iranian nurses should be able to gain a suitable position in the health and nursing policymaking system [104]. They should improve their policymaking knowledge through special educational programs both in undergraduate and higher graduate degrees. Increased exposure to and engagement in policymaking issues during nursing education is one factor that can empower nurses [105]. Therefore, nursing faculties should focus on the preparation and engagement of nursing students into healthcare policymaking discussions [106].

To construct a powerful profession that would be able to defend nurses’ rights, the level of membership by nurses to organizations that can develop a stance in social policymaking and advocate for nurses should be increased. Participation in professional nursing organizations or any association that uses a pluralistic advocacy voice provides networking opportunities and collects resources for exercising collective power. Nurses need to adhere to the same value-oriented principles of nursing and develop a united power source that can influence health and nursing policies using a collective voice [105,107,108].

In addition, INOs and associations should provide opportunities for nurses to enter policymaking initiatives. Nurses can also be engaged directly in policy making by gaining formal leadership positions in the healthcare system and being involved in lobbying and supporting nurse policy makers in professional organizations [95]. In this case, nurses can be a strong voice in health policy formulations.

The poor public image of nursing can withdraw nurses from policy making and social activities, reduce their credibility [39], and reduce their power [109,110]. However, the public image of the nursing profession was improved during the COVID-19 pandemic because the crucial role of nurses in healthcare settings was felt more acutely [111]. Nurse leaders and managers should make most of these new changes in the public image of the nursing profession, try to gain a proper position for nurses in healthcare policy making, and improve the job conditions of nurses.

## 6. Conclusions

Nurses play a key role in the provision of care and optimization of public health, which has become especially prominent during the COVID-19 pandemic. They can provide high-quality healthcare if they receive adequate support and have appropriate QWL and job security. During the current pandemic, the working and employment conditions of many nurses in Iran have been affected due to the imposed insecure employment contracts.

Iranian nurses are at the frontline of the fight against COVID-19 and perform important measures not only for individual health outcomes, but also for public health, including community-wide protection, screening, prevention efforts, and vaccination. Therefore, they need to receive recognition and better support, and their employment based on short-term and inappropriate contracts should be stopped. Healthcare officials, nurse managers, and nurse leaders should increase efforts to ensure that the dignity, QWL, and job security of nurses are preserved and nurses’ capacities to provide high-quality care are maintained. Enforceable standards, along with economic policies designed to considerably promote the employment status of Iranian nurses, are required.

Appropriate participation in healthcare policy making requires an improvement in nurses’ policymaking competencies. Nurses need to improve their awareness and become equipped with the knowledge and skills of policy making and learn how to develop practical strategies that can have a positive impact on nurses’ job conditions. In addition, coalition and lobbying can help to improve the power of nurses to resist insecure job contracts and ensure their dignity and job security. Increasing organizational membership is suggested to build a strong career for nursing advocates. Nurse leaders and managers should make the most of the opportunities created during COVID-19 to participate in policymaking initiatives and improve the current QWL of nurses.

## Data Availability

Not applicable.
